# Retinoid Signaling in Intestinal Epithelial Cells Is Essential for Early Survival From Gastrointestinal Infection

**DOI:** 10.3389/fimmu.2020.559635

**Published:** 2020-10-08

**Authors:** Lindsay M. Snyder, Juhi Arora, Mary J. Kennett, Veronika Weaver, Margherita T. Cantorna

**Affiliations:** ^1^Department of Veterinary and Biomedical Sciences, The Pennsylvania State University, University Park, PA, United States; ^2^Center for Evolutionary & Theoretical Immunology, University of New Mexico, Albuquerque, NM, United States

**Keywords:** vitamin A deficiency, infection, barrier, gastrointestinal tract, retinoic acid receptor

## Abstract

Vitamin A deficiency (A–) increases morbidity and mortality to gastrointestinal (GI) infection. Blocking retinoid signaling (dominant negative retinoic acid receptor, dnRAR) in intestinal epithelial cells (IEC, ^IEC^dnRAR) had no effect on vitamin A absorption, the expression of tight junction proteins or the integrity of the barrier. Immune cells in the gut were present in normal frequencies in the ^IEC^dnRAR mice, with the exception of the T cell receptor (TCR)αβ+/CD8αα cells, which were significantly lower than in wildtype littermates. Challenging the ^IEC^dnRAR mice with dextran sodium sulfate to induce colitis or *Citrobacter rodentium* infection resulted in similar disease to wildtype littermates. Feeding mice vitamin A deficient diets reduced vitamin A status and the A– ^IEC^dnRAR mice developed more severe colitis and *C. rodentium* infection. In particular, retinoid signaling in the IEC was crucial for the A– host to survive early infection following *C. rodentium*. Treating A– mice with retinoic acid (RA) beginning on the day of infection protects most mice from early lethality. However, RA treatment of the A– ^IEC^dnRAR mice was ineffective for preventing lethality following *C. rodentium* infection. Retionid signaling in IEC is critical, especially when there are reduced levels of dietary vitamin A. IEC are direct targets of vitamin A for mounting early defense against infection.

## Introduction

Vitamin A is a micronutrient that is essential for embryonic development, vision, and immune function. Vitamin A deficiency (A–) is a persistent problem in resource limited countries worldwide. It is estimated that over 250 million preschool-age children are vitamin A deficient (1, 2). Vitamin A deficient children develop night blindness and have increased susceptibility to enteric infections (1). Longitudinal studies have shown that supplementing children with vitamin A deficiency with one or two bolus doses of vitamin A reduced infection rates and lessened the severity of enteric infections (3). Vitamin A deficiency is prevalent in developing countries and vitamin A protects the gastrointestinal (GI) tract from infection.

The GI tract is composed of a population of heterogeneous cells whose role is to maintain ignorance of the large number of antigens present in food as well as the abundant microbiota. The mechanisms by which tolerance in the gut is controlled includes complex interactions between the microbiota, the intestinal epithelial cells (IEC) and the immune system. The IEC express tight junction proteins that maintain intestinal integrity and pathogen recognition receptors that function as part of the innate immune response (4, 5). When the barrier becomes compromised following infection, bacteria breach the barrier which can elicit local and systemic inflammation (6, 7). IEC regulate and activate intestinal immunity in the GI tract and are important for resistance to infection and development of inflammatory bowel disease (IBD) (6–8). The IEC of the GI tract are important for the regulation and maintenance of homeostasis.

Vitamin A status regulates intestinal barrier function and is important for the production of the mucous layer that lines the gut. Vitamin A deficiency resulted in keratinization of the mucosa (9, 10). The active metabolite of vitamin A (retinoic acid, RA) induced the expression of several tight junction proteins including: ZO-1, occludin, claudin-6, and claudin-7 (11, 12). Supplementing vitamin A deficient children with vitamin A, decreased urine lactulose/ mannitol levels, suggesting that vitamin A enhances the intestinal barrier integrity in humans (13, 14). Vitamin A is an important regulator of IEC and GI barrier function.

Vitamin A is an essential regulator of the development and function of the immune system. In the GI tract, dendritic cells metabolize vitamin A to produce RA locally (15). RA upregulates the expression of gut-homing receptors α_4_β_7_ and CCR9 receptors on T and B cells (16, 17). Mice with a dominant negative retinoic acid receptor (dnRAR) expressed in T cells failed to clear *Citrobacter rodentium* infection (18). RA treatment suppressed experimental colitis by inhibiting IL-17 and inducing IL-10 and T regs *in vivo* (19). RA induced IL-22 by innate lymphoid cells and γδ T cells that was critical for resolution of inflammation (20). IL-22 induced the production of anti-bacterial peptides by IEC and protected the gut from injury (20, 21). Vitamin A regulates the immune response in the gastrointestinal tract.

We hypothesized that vitamin A was a direct regulator of IEC function and that mice with IEC that were refractory to retinoids would be more susceptible to infectious or chemical injury. To determine the effects of vitamin A on IEC function, mice were generated in which retinoid signaling was inhibited in IEC by expressing the dnRAR in villin expressing cells (^IEC^dnRAR). The dnRAR inhibits retinoid signaling through all 3 RAR (α, β, and γ) receptors (22, 23). The ^IEC^dnRAR mice were not different than the wildtype (WT) littermates in growth rate, vitamin A status, barrier function, and expression of other markers of IEC function. The ^IEC^dnRAR mice had reduced frequencies of T cell receptor (TCR)αβ+/CD8αα+ cells in the gut compared to the WT littermates. Challenging the ^IEC^dnRAR mice with dextran sodium sulfate (DSS) or *C. rodentium* resulted in colitis that was not different than WT. Feeding, the ^IEC^dnRAR mice, vitamin A deficient diets resulted in the increased susceptibility to colitis and severe *C. rodentium* infection. The A– ^IEC^dnRAR mice developed a lethal infection with *C. rodentium* that was refractory to RA treatment. We concluded that IEC are direct targets of vitamin A that are especially critical when the amount of dietary vitamin A is low. In the A+ ^IEC^dnRAR, the role of RA in IEC was compensated for by the effects of vitamin A on other targets in the GI tract.

## Materials and Methods

### Animals

**Figure 1:**
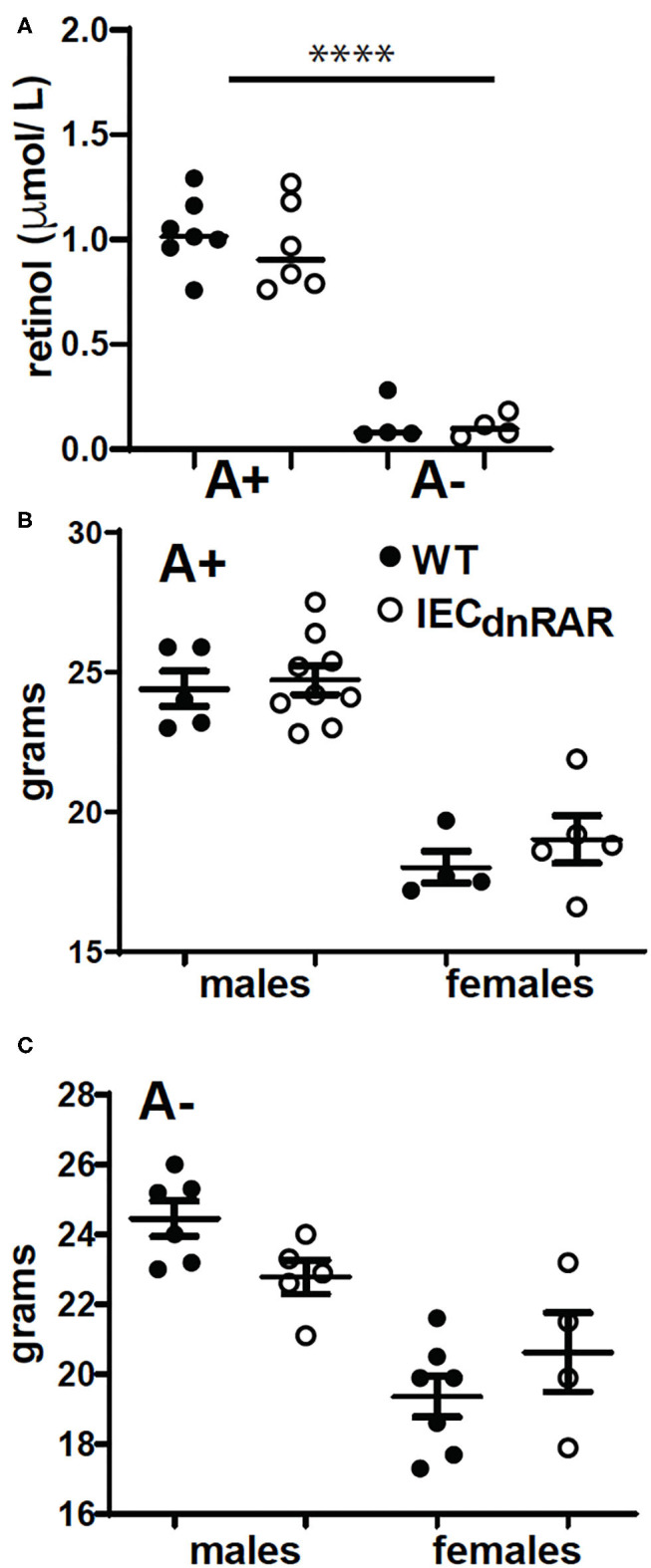
^IEC^dnRAR mice were phenotypically similar to WT mice. For each experiment serum was collected and pooled to monitor vitamin A status. **(A)** Serum retinol values. Values are the mean ± SEM of 4–7 pooled experiments. WT and ^IEC^dnRAR **(B)** A+ and **(C)** A– mice were weighed. Values are the mean ± SEM of *n* = 4–9 mice of each gender. One-way ANOVA with Bonferroni post-test **(A)**, and unpaired student's *T*-test **(B,C)**. ****P < 0.0001.

C57BL/6J villin^Cre+^, Lck^Cre+^, or LysM^Cre+^ mice were originally from Jackson Laboratories (Bar Harbor, ME) and were bred and maintained at the Pennsylvania State University (University Park, PA) according to IACUC and university guidelines. Mice expressing dnRAR^fl/fl^ were generously provided by Dr. Randolph J. Noelle (Dartmouth Medical School, Lebanon, NH). Villin^Cre+^ mice were crossed with dnRAR^fl/fl^ mice to generate ^IEC^dnRAR mice with blocked retinoid signaling through all 3 RAR (α, β, and γ) isoforms in intestinal epithelial cells (22). dnRARfl/fl mice were crossed with Lck^Cre+^ to make ^T^dnRAR and LysM^Cre+^ to make ^LysM^dnRAR mice. dnRAR^fl/fl^ Cre- (WT) littermates were used as controls. A+ and A– mice were generated by feeding pregnant females on purified diets with and without 25 μg/d retinyl acetate as previously described (24). Serum was collected and pooled from each batch of experimental mice to confirm vitamin A status. By 8 wks of age there is a significant difference in serum retinol among the A+ and A– mice (Figure 1A). Vitamin A status was determined by quantifying serum retinol levels using ultra-highpressure liquid chromatography (UPLC). Vitamin A deficiency was defined as serum retinol concentrations <0.7 μmol/L.

### Dextran Sodium Sulfate (DSS)

3.75–4.5% DSS (MP Biomedicals, Solon, OH) was administered orally in the drinking water for 5 days followed by 5 days on water for recovery exactly as described (25–28). The amount of DSS used in the experiments was based on the starting weight of the mice and the use of purified diets that require higher amounts of DSS to induce injury (25–28). None of the mice bled rectally and therefore blood scores were not included in the analyses. Body weight changes were monitored for 10 days. Mice were euthanized at d8 or d10 post DSS exposure. Colon lengths were measured, and distal colon tissue was collected for histological analysis (Supplementary Methods).

### Citrobacter rodentium

Nalidixic acid resistant *C*. *rodentium* strain ICC169 was kindly provided by Gad Frankel (London School of Medicine and Dentistry, London, UK). Bacteria were cultured in Luria-Bertani broth (LB; Becton, Dickinson, & Co, Franklin Lakes, NJ) or LB agar for 18–24 h at 37°C. Overnight log phase bacterial cultures in LB broth were used to prepare inoculums. Adult mice 8–10 wks of age were individually housed, fasted overnight, and then orally gavaged with 100 μl of sterile PBS containing 5 × 10^9^ CFU *C*. *rodentium*. Fecal pellets, spleens, and livers were collected, homogenized and plated in serial dilutions on LB agar plates containing nalidixic acid to quantify bacterial burdens and track infection kinetics as we described (18, 24).

### Flow Cytometry

Single cell suspensions of spleen, meseneteric lymph nodes (MLN) and thymus were made exactly as previously described (29–31). The Peyer's patches were removed and the entire small intestine (SI) IEL and SI or colon IEC were isolated as we have described previously (30, 32, 33). SI and colon tissues were cut longitudinally to increase surface area and incubated twice (20 min at 37°C) with 1 mM 1,4 dithiothreitol (DTT, Sigma Aldrich) and 10 mM EDTA to release IEC and IEL (30, 32, 33). A 25/40% discontinuous Percoll (Sigma Aldrich) gradient was used to purify IEC while a 40/80% discontinuous gradient was used to purify IEL. 1–2 million cells were stained with fluorescein isothiocyanate (FITC) CD8β, FITC or phycoerythrin (PE) GL3 (γδ TCR), PE-CF594 CD4, PE-Cy 5 TCRβ, Brilliant violet (BV) 421 TLA (BD Biosciences, San Jose, CA), PE IL15Rα (Thermo Fisher Scientific, Waltham, MA), PE-Cy7 TLR4, or PE-Cy7 CD8α (BioLegend, San Diego, CA). CD8αα was detected in the thymus with PE-labeled TL-tetramer (T3b) (34). The tetramers were a gift from Dr. Hilde Cheroutre (La Jolla Institute for Allergy and Immunology, La Jolla, CA). Single positive and fluorescence minus one (FMO) controls were used to set gates. Cells were analyzed on an FC500 benchtop cytometer (Beckman Coulter, Brea, CA) or a Becton Dickinson LSR Fortessa cytometer (Becton, Dickinson and Company, Franklin Lakes, NJ), and data was analyzed using FlowJo 7.6.5 software (Tree Star, Ashland, OR).

### FITC Dextran Permeability Assay

4 kDa FITC dextran (Sigma Aldrich, St. Louis, MO) permeability assay was performed as previously described (26). Briefly, mice were fasted for 4 h and then gavaged with an 80 mg/kg dose of FITC dextran. 4 h later, mice were bled to obtain serum and a FITC dextran standard curve (20 to 0.3125 μg/ml) was prepared by diluting the stock solution with PBS. The standard and serum samples from naïve and DSS treated mice were transferred to black bottom 96 well plates and fluorescence was read at 525 nm on a Perkin Elmer Wallace Microplate Reader (GMI, Ramsey, MN). A linear curve was fitted to the standard and used to quantify serum FITC dextran levels.

### Histology

Distal colons from naïve and *C*. *rodentium* infected mice were fixed in 4% formalin, embedded in paraffin, sectioned, and stained with hematoxylin and eosin at the Pennsylvania State University Animal Diagnostics Laboratory. *C*. *rodentium* tissue sections were coded and scored by a board-certified laboratory animal veterinarian with pathology training (Dr. Mary Kennett, University Park, PA). Tissue samples were scored for inflammatory cell infiltrate cell infiltrates and the severity of mucosal damage, edema, and crypt loss (35). The five longest crypts were identified visually from each sample and measured to determine an average crypt length.

### RT-PCR

Tissues were snap frozen and stored at −80°C until RNA isolation with TriZOL (Invitrogen, Carlsbad, CA). Cells (2 × 10^6^) were suspended in 0.5 mL TriZOL and stored at −80°C until processing. RNA isolation was performed according to TriZOL manufacturer's protocol. Complementary DNA (cDNA) was created by reverse transcribing 2–4 ug RNA using TaqMan reverse transcription kit (Applied Biosystems, Carlsbad, CA). qPCR was performed using SYBR green mix (BioRad, Hercules, CA) and the MyiQ Single-Color Real Time PCR machine (BioRad). Relative standards were prepared by serially diluting DNA products of the genes of interest. A standard curve was generated to quantify relative expression levels in samples. Relative expression levels were normalized to a housekeeping gene (HPRT) and fold change values were reported relative to WT or untreated control tissues. Primers are listed in Supplementary Table 1.

### Statistical Analyses

Statistical analyses were performed using GraphPad Prism (GraphPad, La Jolla, CA). Two-tailed student *t*-tests were used to compare gene expression fold change, histological scores, and DSS parameters. A student's *t-*test with Welch's correction was also used to compare data sets with unequal variances and a Mann Whitney test was used to compare data sets that were not normally distributed. One-way analysis of variance (ANOVA) with Bonferroni's *post hoc* test was used to analyze histology scores. Two-way ANOVA with Bonferroni's *post hoc* test was used to analyze IEL/thymocyte populations through time and fecal shedding and weight loss curves. Log rank Mantel-Cox test was used to assess survival rates between genotypes and treatments. A *P* < 0.05 was used as the threshold to determine statistically significant changes.

## Results

### Normal Intestinal Epithelial Cells in ^IEC^dnRAR Mice

Genotype had no effect on body weight in male or female mice that were either A+ (Figure 1B) or A– (Figure 1C). A+ ^IEC^dnRAR and A+ WT mice had significantly higher serum retinol than age matched A– ^IEC^dnRAR and A– WT mice (Figure 1A). There were no differences in serum retinol as a result of genotype (Figure 1A). The total number of lymphocytes in thymus, spleen, MLN and SI IEL were not different in the ^IEC^dnRAR and WT mice (Supplementary Figure 1). The frequencies of CD4+ cells in the MLN and the frequencies of CD19+ cells in the spleen were slightly but significantly lower in the ^IEC^dnRAR as compared to the WT mice (Supplementary Figure 1). The number of IEC cells from ^IEC^dnRAR and WT mice were not different in either the SI or colon (Supplementary Figure 2A). The frequency of IEC that express TLR-4, IL-15Rα, and TLA were the same in ^IEC^dnRAR and WT SI and colon (Supplementary Figure 2). In addition, mean fluorescence intensities (MFI) for TLR4, MHCI, IL15R-α, and TLA were the same on the WT and ^IEC^dnRAR IEC (Supplementary Figure 2 and data not shown). The expression of mRNA for *madcam1, ccl25, occludin, claudin 6, and claudin 7* were not different in ^IEC^dnRAR and WT mice (Supplementary Figure 3). The IEC from ^IEC^dnRAR mice were phenotypically similar to WT IEC.

### Fewer CD8αα Expressing T Cells in ^IEC^dnRAR Mice

**Figure 2:**
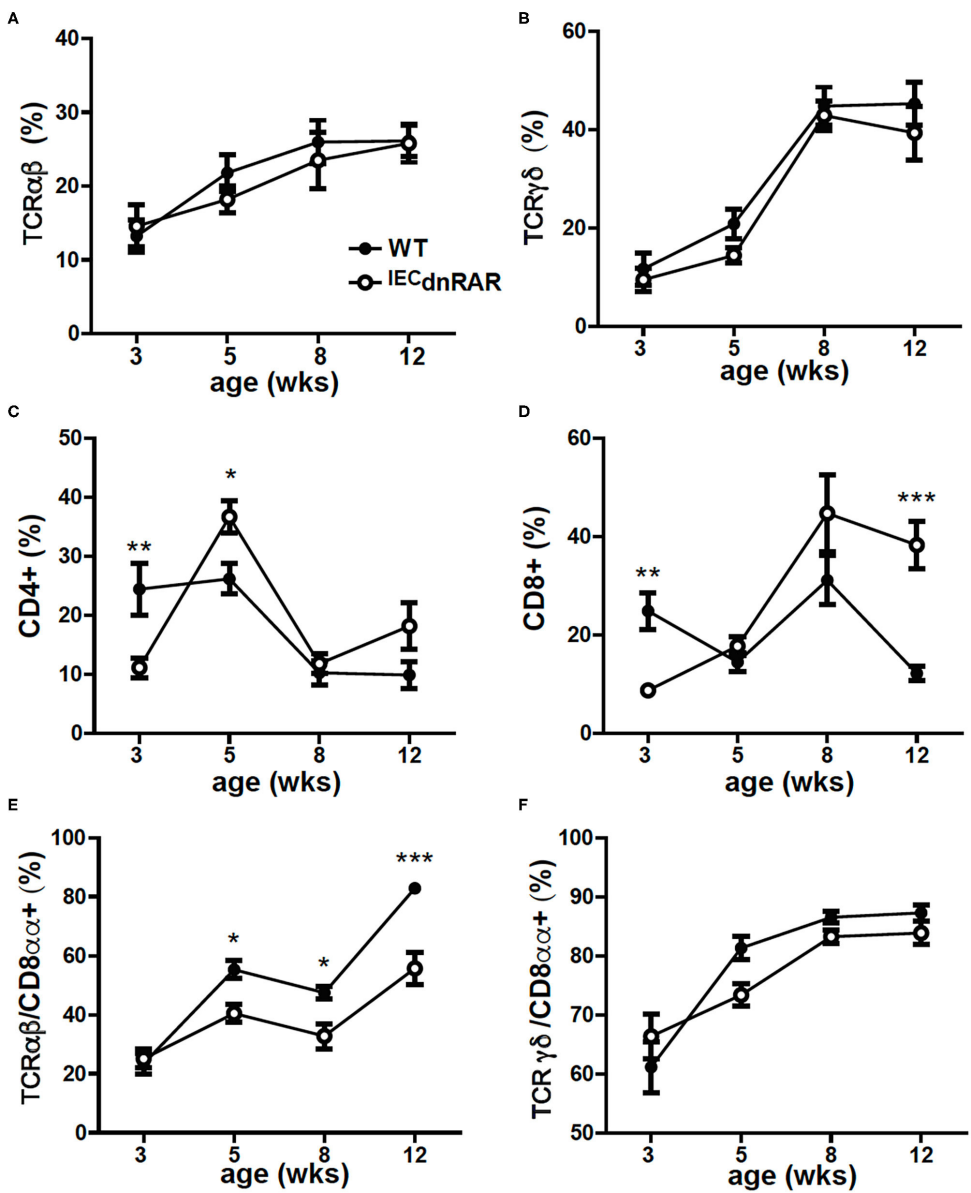
Reduced frequencies of CD8αα+ T cells in the SI of A+ ^IEC^dnRAR mice. The A+ WT and A+ ^IEC^dnRAR frequencies of **(A)** TCRαβ+, **(B)** TCRγδ+, **(C)** CD4+, **(D)** CD8+, **(E)** TCRαβ+/CD8αα+, and **(F)** TCRγδ+/CD8αα+ T cells in the SI IEL. Values are the mean ± SEM of two combined experiments with *n* = 7–11 mice at each time point. Two-way ANOVA with Bonferroni post-test, *P < 0.05, **P < 0.01, ***P < 0.001.

**Figure 3:**
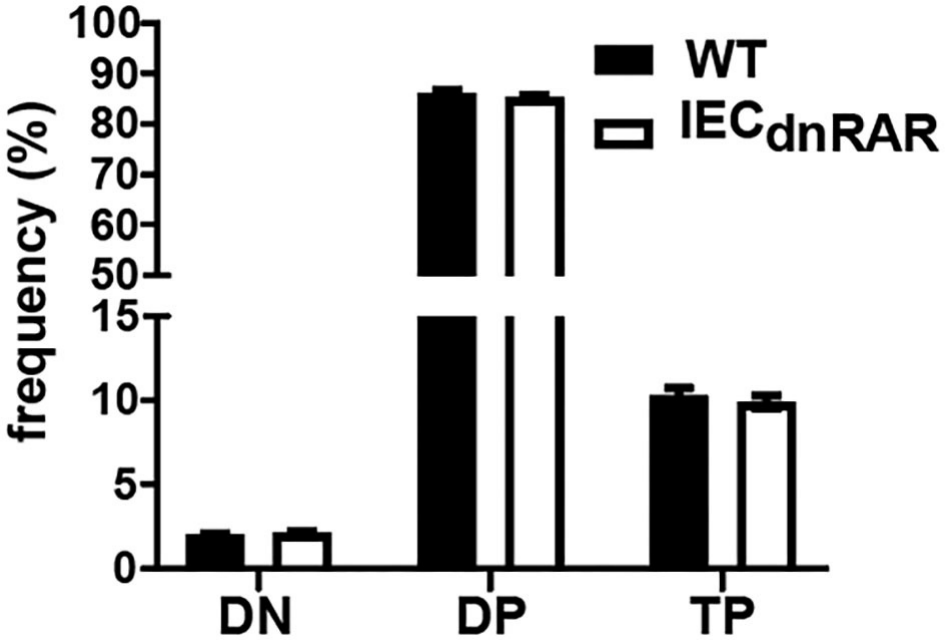
Normal thymocyte development in ^IEC^dnRAR mice. The frequencies of DN, DP, and TP cells in the thymus of WT and ^IEC^dnRAR mice. Values are mean ± SEM of two combined experiments, *n* = 6–11 mice of each genotype. Statistical significance was evaluated using two-way ANOVA with Bonferroni post-test.

IECs are important in the development of intestinal T cell populations. The total number of cells isolated from the SI IEL were the same in A+ ^IEC^dnRAR and WT mice (Supplementary Figure 1). The frequencies of TCRαβ+ T cells, CD4+, and TCRγδ+ T cells in the IEL of ^IEC^dnRAR and WT were the same in adult mice (Figures 2A–C). There were some significant shifts in CD4+ and CD8+ T cell frequencies in SI IEL as the mice aged (Figures 2C,D). At 8 wks of age there were no differences in CD4+ or CD8+ frequencies but at 12 wks the CD8+ T cells in ^IEC^dnRAR mice were significantly higher compared to WT mice (Figure 2D). The TCRαβ+/CD8αα+ cells were lower in the ^IEC^dnRAR mice beginning at 5 wks of age and continuing to adulthood (Figure 2E), while the TCRγδ+/CD8αα+ cells were not different in WT and ^IEC^dnRAR mice (Figure 2F). All TCRαβ+ T cells, including the TCRαβ+/CD8αα+ cells, develop in the thymus. In order to determine whether the ^IEC^dnRAR had a defect in thymocyte development, thymic precusors were measured in ^IEC^dnRAR and WT mice. Double negative (DN, CD4–/CD8–) cells become double positive (DP, CD4+/CD8+) cells that then develop into single CD8+ or CD4+ T cells (30, 34, 36). A subset of the DP thymocytes become triple positive (TP, CD4+/CD8+/CD8αα) and then downregulate all three receptors as they exit the thymus (34). TP cells are the thymic precursors of TCRαβ+/CD8αα+ cells in the SI (34). The frequencies of the DN, DP, and TP cells in the thymus were not different in ^IEC^dnRAR and WT mice (Figure 3), ruling out a thymus development effect of the ^IEC^dnRAR. IL-15, IL-15 receptor (R), TLA and TGF-β are important in the induction of CD8αα on TCRγδ+ and TCRαβ+ T cells in the gut (37). *Il15r*α*, Tla, and Tgf*β mRNA expression was the same in ^IEC^dnRAR and WT SI (Supplementary Figure 4). *Il15* mRNA expression was significantly higher in ^IEC^dnRAR SI than in WT (Supplementary Figure 4). The reduced frequency of TCRαβ+/CD8αα+ in ^IEC^dnRAR SI was not due to changes in IL-15, IL-15Rα, TLA, or TGF-β. Expression of the dnRAR in IEC resulted in fewer TCRαβ+/CD8αα+ in the SI as compared to WT mice.

### WT and ^IEC^dnRAR Mice Are Resistant to DSS Colitis

**Figure 4:**
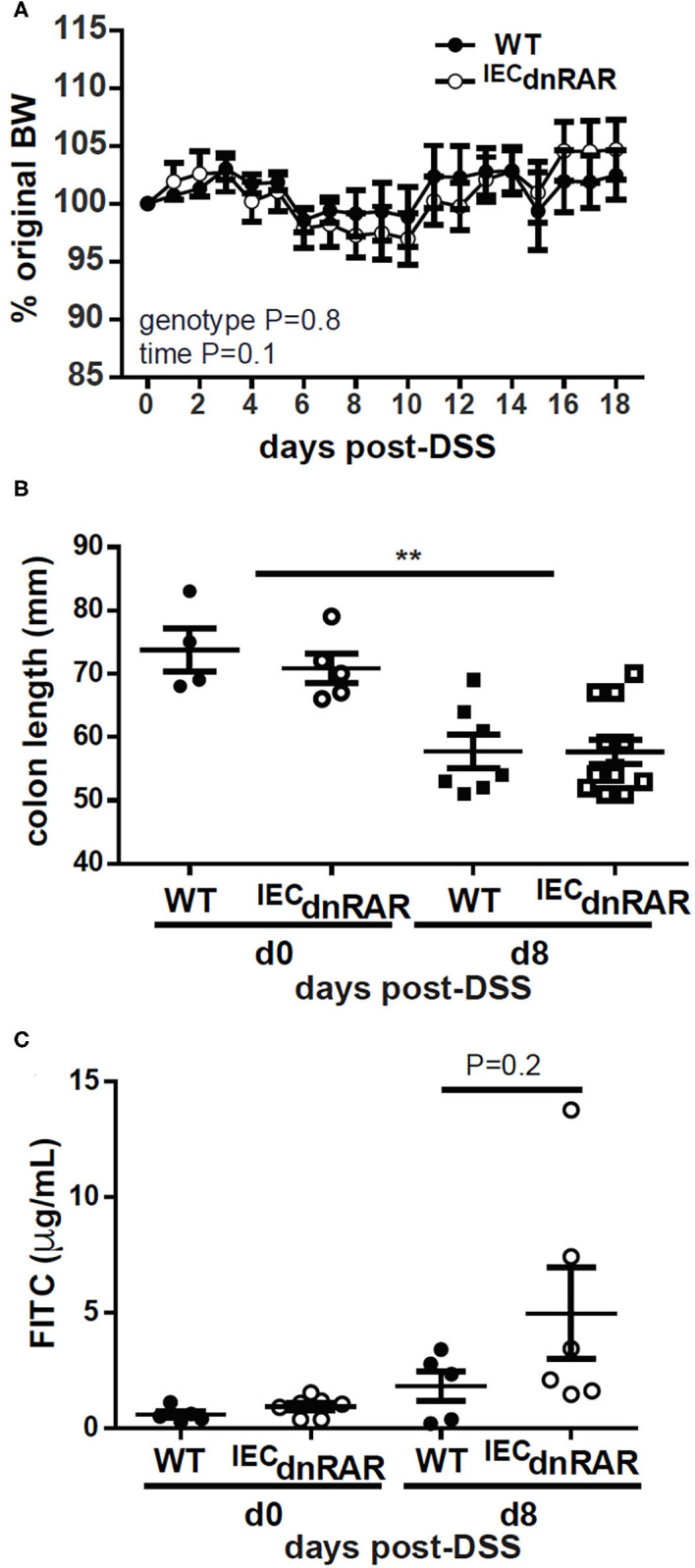
No change in susceptibility to DSS in A+ ^IEC^dnRAR mice. **(A)** Weight loss, **(B)** colon length, and **(C)** intestinal permeability following DSS administration. Values are the mean ± SEM of two experiments with *n* = 6–12 mice her group and time point. Two-way ANOVA with Bonferroni post-test **(A)**, and one-way ANOVA **(B,C)**, **P < 0.01.

TCRαβ+/CD8αα+ T cells protect the host from GI injury (38). Vitamin A sufficient (A+) WT and ^IEC^dnRAR mice were treated with DSS to induce colitis. DSS induced weight loss in A+ WT and ^IEC^dnRAR mice, when the males and females were analyzed separately (significant time effect, Supplementary Figure 5A), but not when they were combined together (Figure 4A). There was no genotype effect on weight loss following DSS treatment in males, females or combined males and females (Supplementary Figure 5A and Figure 4A). After 8 days of DSS treatment A+ WT and ^IEC^dnRAR treated mice had significantly shortened colon lengths compared to day 0 colon length (Figure 4B). There were no differences between the colon lengths of A+ WT and ^IEC^dnRAR mice at day 0 or day 8 post-DSS (Figure 4B). There was a small but insignificant increase in intestinal permeability that occurred following DSS treatment of A+ WT and ^IEC^dnRAR mice (Figure 4C). Histopathology sections were not different in the A+ WT and ^IEC^dnRAR mice at day 10 post-DSS (Supplementary Figures 5C, 6A). There was no effect of ^IEC^dnRAR expression on the susceptibility of mice to DSS.

**Figure 5:**
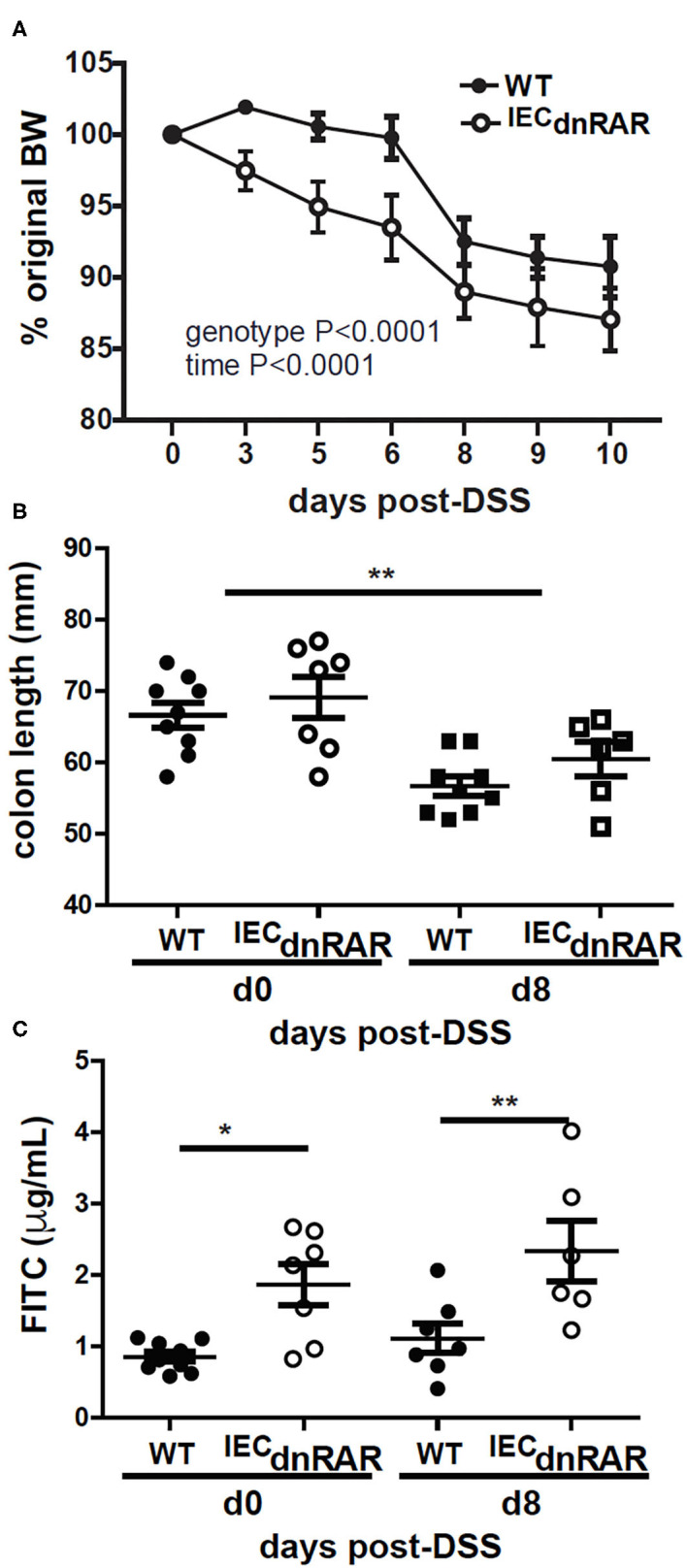
Increased susceptibility to DSS in A– ^IEC^dnRAR mice. **(A)** Weight loss, **(B)** colon length, and **(C)** intestinal permeability following DSS treatment of A– WT and A– ^IEC^dnRAR mice. Values are mean ± SEM of two experiments with *n* = 6–9 mice per group. Two-way ANOVA with Bonferroni post-test **(A)** or one-way ANOVA **(C,D)** was used to determine significance, *P < 0.05, **P < 0.01.

Vitamin A deficient (A–) WT and ^IEC^dnRAR mice were also challenged with DSS. A– ^IEC^dnRAR mice lost significantly more weight following DSS treatment than their A– WT counterparts (Figure 5A). The effect of genotype on A– weight loss was largely due to the males and not the females since there was no effect of genotype on weight loss in the A– females (Supplementary Figure 5B). All other parameters of colitis severity were unaffected by sex. As expected colon lengths were shorter at day 8 post-DSS than at day 0 (Figure 5B). There was no effect of genotype on A– colon length either at day 0 or day 8 post-DSS (Figure 5B). Colonic histopathology at d10 post-DSS was not different in A– WT and A– ^IEC^dnRAR mice (Supplementary Figures 5C, 6A). Intestinal permeability was significantly higher in A– ^IEC^dnRAR mice compared to A– WT mice at both day 0 and day 8 post-DSS (Figure 5C). Expression of the dnRAR in IEC cells, with vitamin A deficiency, resulted in the increased permeability of the GI tract, and the increased weight loss following injury of the GI tract with DSS.

### A– ^IEC^dnRAR Develop a Severe Early Infection With *Citrobacter rodentium*

**Figure 6:**
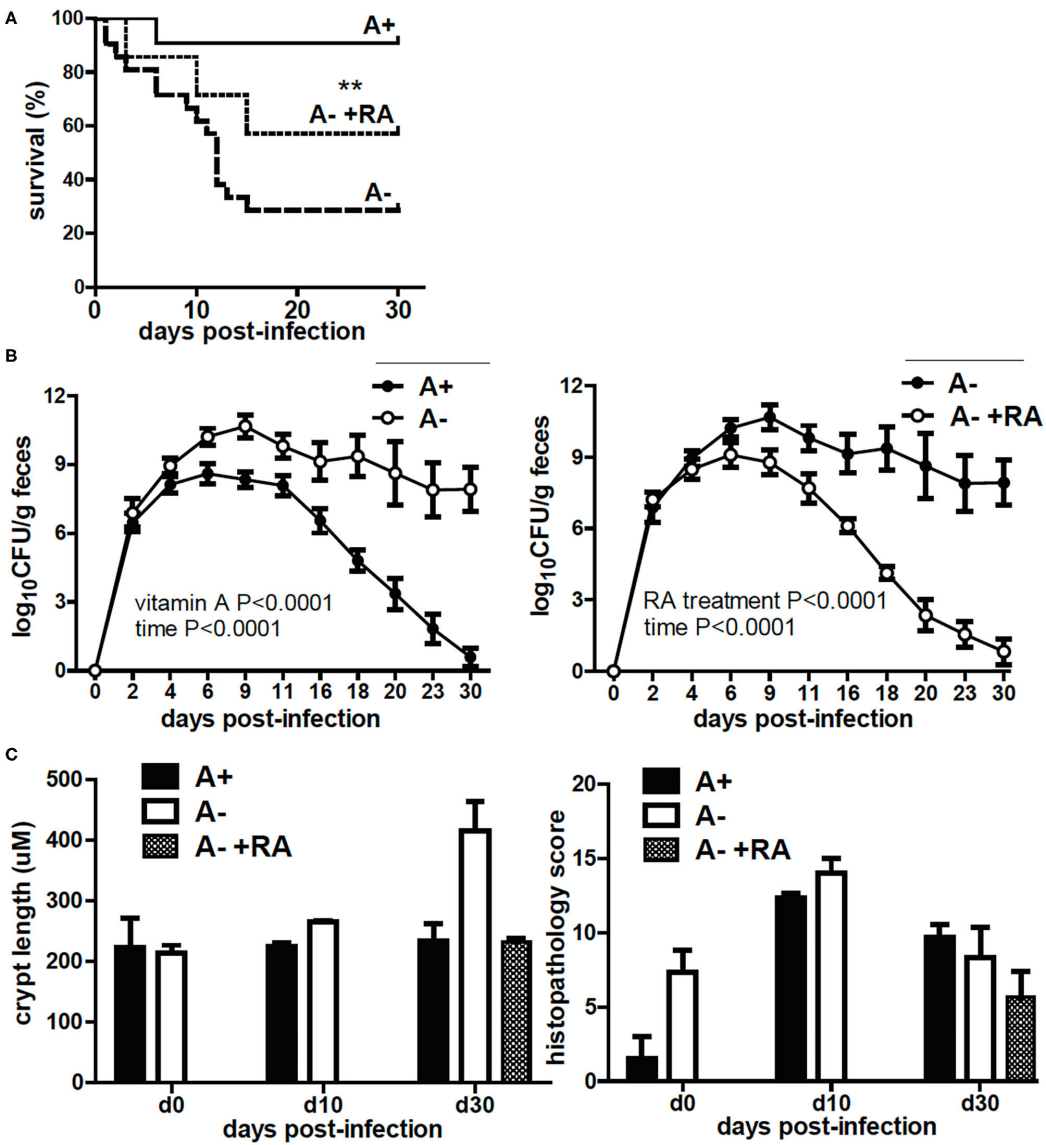
C. *rodentium* infection in A+ ^IEC^dnRAR, A– ^IEC^dnRAR mice and A– +RA ^IEC^dnRAR mice. A+ ^IEC^dnRAR, A– ^IEC^dnRAR and A– +RA ^IEC^dnRAR mice were infected with *C. rodentium* and **(A)** survival, **(B)** fecal shedding, and **(C)** crypt length/histopathology score of the distal colon were measured. Values are the mean ± SEM of *n* = 4–10 mice per group and time-point. Significance was determined using the Log rank Mantel-Cox test **(A)**, two-way ANOVA with Bonferroni post-test **(B)**, and one-way ANOVA **(C)**, **P < 0.01.

A– mice had reduced survival following *C. rodentium* infection (24). The surviving A– mice were unable to clear the infection (24). Treating A– mice with RA on the day of infection or at the peak of infection completely protected the mice and resulted in clearance of *C. rodentium* (18). Three groups of ^IEC^dnRAR mice were infected with *C. rodentium*, A+, A–, and A– +RA treated (Figure 6). The A+ ^IEC^dnRAR mice had *C. rodentium* shedding curves (Figure 6A) that were the same as A+ WT (18, 24). A– ^IEC^dnRAR mice had decreased survival following *C. rodentium* infection (Figure 6A and Table 1). Surviving A– ^IEC^dnRAR mice became chronic shedders of *C. rodentium* and had very high numbers of bacteria in their feces on day 30 post-infection, when A+ ^IEC^dnRAR mice had already cleared the infection (Figure 6B). RA treatment of A– ^IEC^dnRAR mice significantly reduced the fecal shedding of *C. rodentium* and the kinetics of the clearance were similar to the A+ ^IEC^dnRAR mice (Figure 6B). The chronically infected A– ^IEC^dnRAR mice had short, visibly thickened colons. At day 30 post-infection the A– ^IEC^dnRAR mice had significantly longer crypt lengths than all other groups (Figure 6C). The histopathology scores showed increased damage of the A+ and A– colons at day 10 post-infection that resolved by day 30 post-infection (Figure 6C and Supplementary Figure 6B). There were no differences in histopathology scores when comparing A+ ^IEC^dnRAR and A– ^IEC^dnRAR mice (Figure 6C and Supplementary Figure 6B). Expression of the ^IEC^dnRAR exacerbated the effects of vitamin A deficiency on GI infection.

**Table 1:** A– ^IEC^dnRAR are refractory to the early protective effects of RA.

**Genotype**	**WT**		^****LysM****^**dnRAR**		^****IEC****^**dnRAR**	
	**A–**	**A– +RA^a^**	**A–**	**A– +RA**	**A–**	**A– +RA**
Mortality^b^ (*n*)	59% (17)	9% (11)	50% (16)	0% (15)	71% (21)	43% (7)
Chronic^c^ (*n*)	100% (10)	0% (11)	88% (8)	42% (12)	100% (6)	0% (4)

a *RA dosing was started on the day of infection*.

b *Fisher exact test was used to determine the effect of RA treatment on mortality and chronic shedding. There was a significant effect of RA treatments on mortality of A– WT and A– ^LysM^dnRAR mice but not A– ^IEC^dnRAR mice. There was a significant effect of RA on chronic infection rates in A– WT and A– ^IEC^dnRAR mice but not A– ^LysM^dnRAR mice*.

c *Mice were continuing to shed >3 logs of bacteria in the feces on day 37 post-infection*.

### IEC Are Critical Retinoid Targets for Protection From Early Lethality Following *C. rodentium* Infection

Treating A– WT mice with RA at d0 protected mice from lethality and resulted in the complete clearance of *C. rodentium* infection (24) (Table 1). Virulence of *C. rodentium* is regulated by the LEE pathogencity island that includes the transcriptional regulator ler (39). Ler expression is highest at the peak of infection (day 5–7) and then is downregulated (40). *C. rodentium* from A– mice expressed more ler than the *C. rodentium* from RA treated A– mice (Supplementary Figure 7). At day 5 post-infection when both the A– and A– +RA ^LysM^dnRAR had very high infections, ler was lower in the RA treated *C. rodentium* (Supplementary Figure 7). RA treatment of mice that express the dnRAR in macrophage and neutrophil (^LysM^dnRAR) protected the mice from lethality but resulted in chronically *C. rodentium* infected mice (18) (Table 1). At d37 post-infection the A– ^LysM^dnRAR still had *C. rodentium* that expressed ler (Supplementary Figure 7). RA dosing of A– ^IEC^dnRAR mice starting at d0 of infection failed to significantly improve survival rates (Table 1). However, the surviving ^IEC^dnRAR A– +RA mice cleared the *C. rodentium* infection (Table 1 and Figure 6). Functional retinoid signaling in macrophage/neutrophil is not required to survive the early infection but is required for clearance of the infection. Conversely, IEC are critical retinoid targets that prevent early lethality following *C. rodentium* infection.

## Discussion

Mice with a dnRAR expressed in IEC were phenotypically normal and had IEC that expressed similar levels of several tight junction proteins as their WT littermates. Conversely, the ^IEC^dnRAR mice had reduced frequencies of TCRαβ+/CD8αα+ T cells in the IEL. The thymic precursors of the CD8αα+/TCRαβ+ were unaffected by the ^IEC^dnRAR expression. IL-15 and trans-presentation of IL-15 is required for CD8αα maturation and survival (41). IL15R knockout (KO) mice had reduced TCRαβ+/CD8αα+ frequencies and restoration of IL15-Rα expression on IEC restored TCRαβ+/CD8αα populations (41, 42). IL15-Rα expression was normal in ^IEC^dnRAR mice. Other potential regulators of IEL include RA, TLA, TGF-β, IFN-γ, and IL-27 (43, 44). TGF-β and TLA expression were normal in the ^IEC^dnRAR GI tract. IEC have been shown to be required for the production of serum amyloid A (SAA) proteins that are hypothesized to deliver retinol to T cells and IEC important for GI immunity (45). It is possible that the IEC require functional RAR to induce SAA delivery of retinol for the RA mediated induction of CD8αα+ on TCRαβ+ T cells in the gut. There is a cell intrinsic requirement for functional RARs in the IEC to indirectly induce CD8αα+ on TCRαβ+ T cells in the gut.

Expression of the dnRAR in IEC affects the intestinal barrier. Intestinal barrier function, susceptibility to DSS colitis, and resistance to *C. rodentium* infection was not affected by expression of the ^IEC^dnRAR in vitamin A suffient mice. Expression of mRNA for tight junction proteins and intestinal permeability were not different in A+ ^IEC^dnRAR and WT mice (Supplementary Figure 3 and Figure 4). A limitation of the study is that protein levels for the tight junction proteins were not measured. Gattu et.al showed that A+ ^IEC^dnRAR had normal barrier function (45). However, *Salmonella* infection of A+ ^IEC^dnRAR mice resulted in more translocation of bacteria to the spleen and liver than in the WT littermates (45). Blocking retinoid signaling in IEC had mild effects on susceptibility to colitis or protection from GI infection. When the ^IEC^dnRAR mice were vitamin A deficient, blocking retinoid signaling in IEC had severe consequences. Intestinal permeability and weight loss were significantly higher in A– ^IEC^dnRAR mice compared to A– WT mice (Figure 5). Vitamin A is a critical regulator of the GI barrier and blocking the effects of retinoids in IEC cells resulted in increased permeability even when the mice were unchallenged (Figure 5). A– ^IEC^dnRAR mice were more susceptible to DSS colitis (Figure 5) and extremely susceptible to *C*. *rodentium* (72% mortality rate, Figure 6). Treating A– WT and A–^LysM^dnRAR mice with RA on the day of infection eliminated the lethality of a *C. rodentium* infection [Table 1 (18, 24)]. RA treatments were ineffective for preventing mortality in the *C. rodentium* infected A– ^IEC^dnRAR mice (Table 1). There was no lethality in the A+ ^IEC^dnRAR or the A+ ^LysM^dnRAR infected with *C. rodentium* [Figure 6 (18)]. When vitamin A is low IEC must be able to respond to RA, which helps to maintain the barrier and protect the host from early lethality following GI infection. The early lethality following *C. rodentium* infection in A– mice requires retinoid signaling in IEC but not macrophage/neutrophils.

The inflammation following *C. rodentium* infection has been shown to be greatest at the peak of infection, followed by resolution of inflammation as the infection is cleared. Infection of germfree mice with *C. rodentium* resulted in inflammation that was highest at 10–14 days post-infection and then resolution of inflammation; although the mice remained colonized with high numbers of organisms (40). Ler was downregulated in the chronically *C. rodentium* monocolonized mice without inflammation (40). Chronically infected A– and A– +RA ^LysM^dnRAR mice shed high numbers of *C. rodentium* in the feces 30 days post-infection [Table 1 (18, 24)]. RA treatment of the A– ^LysM^dnRAR on the day of *C. rodentium* infection, protected the mice from early lethality but 42% of the mice developed chronic infection (Table 1). RA treated A– ^LysM^dnRAR mice had *C. rodentium* with lower expression of ler at day 5 and day 37 post-infection. Ler expression did not correspond with the ability of the host to eliminate *C. rodentium*, since the RA treated A– ^LysM^dnRAR developed chronic infections (Table 1). RA treatments inhibit the expression of the *C. rodentium* ler pathogenicity genes in otherwise A– hosts perhaps by reducing inflammation (39).

Vitamin A deficiency remains a public health concern in resource limited countries. The GI tract needs vitamin A for mucous production and the regulation of the barrier. During vitamin A sufficiency other cells in the GI tract can compensate for the IEC that express dnRAR to regulate the barrier. Conversely, the development of CD8αα+/TCRαβ+ T cells require IEC with functional retinoid receptors. RA in the IEC protects the vitamin A deficient host from severe GI disease including lethal bacterial infections. It would be important for developing countries to continue to work toward strategies to improve vitamin A nutrition. Even in the vitamin A deficient host, short-term RA treatments restored IEC function protecting the host from the most serious consequences of vitamin A deficiency.

## Data Availability Statement

The raw data supporting the conclusions of this article will be made available by the authors, without undue reservation.

## Ethics Statement

The animal study was reviewed and approved by The Pennsylvania State University IACUC committee.

## Author Contributions

LS, JA, and MC conceptualized and designed the experimental studies. LS, JA, MK, and VW performed the experiments and acquired and analyzed the data. LS and JA drafted the manuscript with the help of MC. All authors approved the publication of the manuscript.

## Conflict of Interest

The authors declare that the research was conducted in the absence of any commercial or financial relationships that could be construed as a potential conflict of interest.
