# Supplementary material for: Retinoid Signaling in Intestinal Epithelial Cells Is Essential for Early Survival From Gastrointestinal Infection
Source: Front Immunol. 2020 Oct 8;11:559635. doi: 10.3389/fimmu.2020.559635 (PMC7578243; doi:10.3389/fimmu.2020.559635)
Supplement: Supplementary file 1 [file Data_Sheet_1.pdf]

Supplemental Methods

Histology. Distal colons from naïve and dextran sodium sulfate (DSS) treated mice were fixed in 4% formalin, embedded in paraffin, sectioned, and stained with hematoxylin and eosin at the Pennsylvania State University Animal Diagnostics Laboratory. Tissue sections were coded and scored by a board-certified laboratory animal veterinarian with pathology training (Dr. Mary Kennett, University Park, PA). DSS scoring used four measures of pathology: inflammatory cell infiltrates-severity (0-4), inflammatory cell infiltrates-extent (0-3), mucosal architecture damage (0-4) and edema-goblet cell loss (0-4) and a total score of 0-15 (Cooper et al., 1993;Erben et al., 2014).

***C. rodentium* ler expression.** *C. rodentium* ler expression was measured in the cecal contents. RNA was isolated from cecal contents (snap frozen) using RNeasy PowerMicrobiome Kit as per manufacturer's instructions (Qiagen, Germantown, MD). Complementary DNA (cDNA) was synthesized by reverse transcribing 1 μg RNA using AMV Reverse Transcription Kit (Promega, Madison, WI). Relative expression levels were normalized to 16SrRNA expression. qPCR was performed using SYBR green mix (BioRad, Hercules, CA) and StepOne Plus Real Time PCR machine (Applied Biosystems, Carlsbad, CA). Gene expression was calculated using comparative CT method (delta delta CT), relative to uninfected mice. Primers are listed in Supplementary (S) Table 1.

References:

Cooper, H.S., Murthy, S.N., Shah, R.S., and Sedergran, D.J. (1993). Clinicopathologic study of dextran sulfate sodium experimental murine colitis. *Lab Invest* 69**,** 238-249.Erben, U., Loddenkemper, C., Doerfel, K., Spieckermann, S., Haller, D., Heimesaat, M.M., Zeitz, M., Siegmund, B., and Kuhl, A.A. (2014). A guide to histomorphological evaluation of intestinal inflammation in mouse models. *Int J Clin Exp Pathol* 7**,** 4557-4576.

Supplemental Table 1: RT-PCR Primers
Hprt
F: 5’- CAGACTGAAGAGCTATTGTAATG-3’
R: 5’- CCAGTGTCAATTATATCTTCCAC-3’

Il15
5’-CAG AGG CCA ACT GGA TAG ATG-3’
5’-ACT GTC AGT GTA TAA AGT GGT GTC AAT-3’

Il15rα
5’-TTG GGA GAG AAA GCT TCT GG-3’
5’-CCA GTG CCA ACA GTA GTG ACA-3’

Tla
5’-AAA AAG ACA CAG GAG TGC ACA G-3’
5’-TGA TGT CAG CAG GGT AGA AGC-3’

Madcam1
5’-GGG CAG GTG ACC AAT CTG TA-3’
5’-ATA GGA CGA CGG TGG AGG A-3’

Ccl25
5’-GAG TGC CAC CCT AGG TCA TC-3’
5’-CCA GCT GGT GCT TAC TCT GA-3’

Occludin
5’-GTC CGT GAG GCC TTT TGA-3’
5’-GGT GCA TAA TGA TTG GGT TTG-3’

Claudin7
5’-GAC GCC CAT GAA CGT TAA GTA-3’
5’-GGA CAG GAG CAA GAG AGC A-3’

Claudin6
5’-TAT CCT GTC CCA GTC CCA AG-3’
5’-GTG CGT CTG TCC TGT GAG TTA C-3’

Ler
5’-AAT ATA CCT GAT GGT GCT CTT G-3’
5’-TTC TTC CAT TCA ATA ATG CTT CTT-3’

16S rRNA
5’-ACT CCT ACG GGA GGC AGC AG-3’
5’-ATT ACC GCG GCT GCT GG-3’

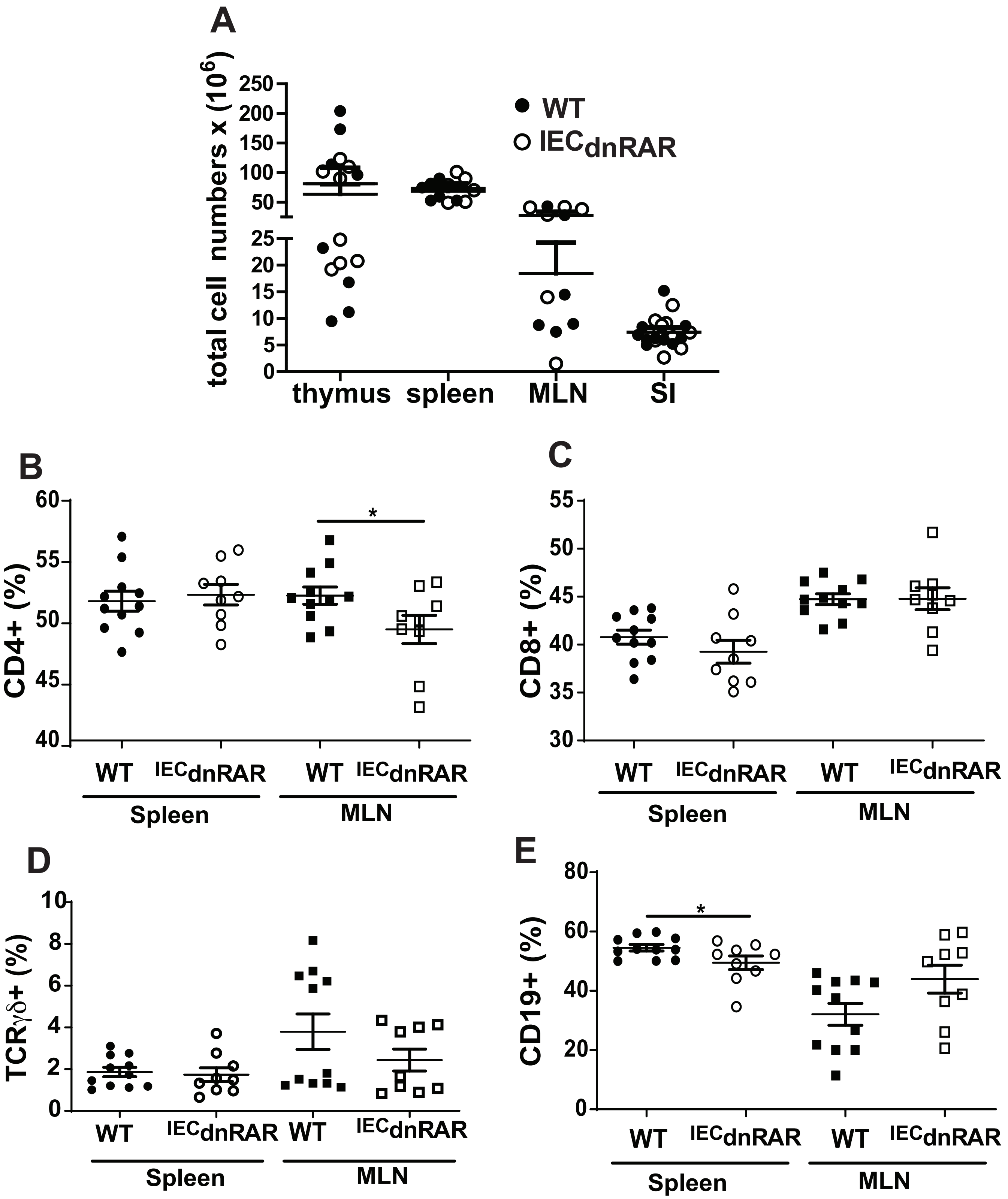

Supplemental Figure 1 A) The total cell numbers isolated from the thymus, spleen, mesenteric lymph node (MLN) and small intestine (SI)of WT and IECdnRAR mice. The frequencies of **B)** CD4, **C)** CD8, **D)** TCRγδ+ and **E)** CD19+cells in the spleen and MLN of WT and IECdnRAR mice. Values are mean ± SEM. n=6-11 mice of each genotype. Statistical significance was evaluated using two way ANOVA with Bonferoni post-tests (A) or using Student's t-test (B-E), *P<0.0.5.

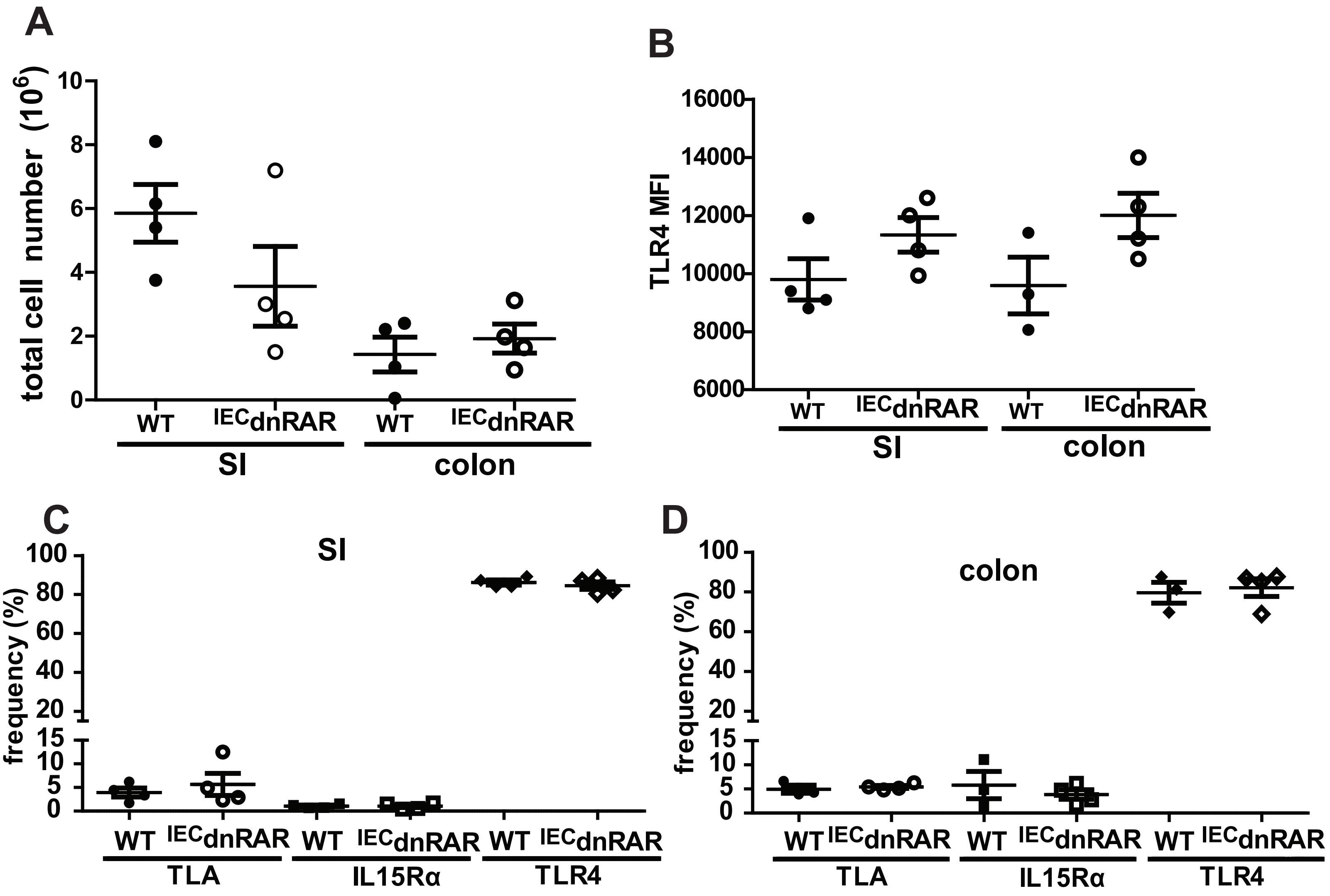

Supplementary Figure 2: Characterization of the SI and colon IEC. A) total IEC cell numbers isolated. **B)** TLR 4 mean fluorescence intensity (MFI), and the frequencies of TLA, IL15Rα, and TLR4 positive cells in the **C)** colon and **D)** colon of WT and ^{IEC}dnRAR mice. Values are mean ± SEM of one experiment and n=4/ group. Mann Whitney test were used to determine there were no differences between genotypes for any measurement.

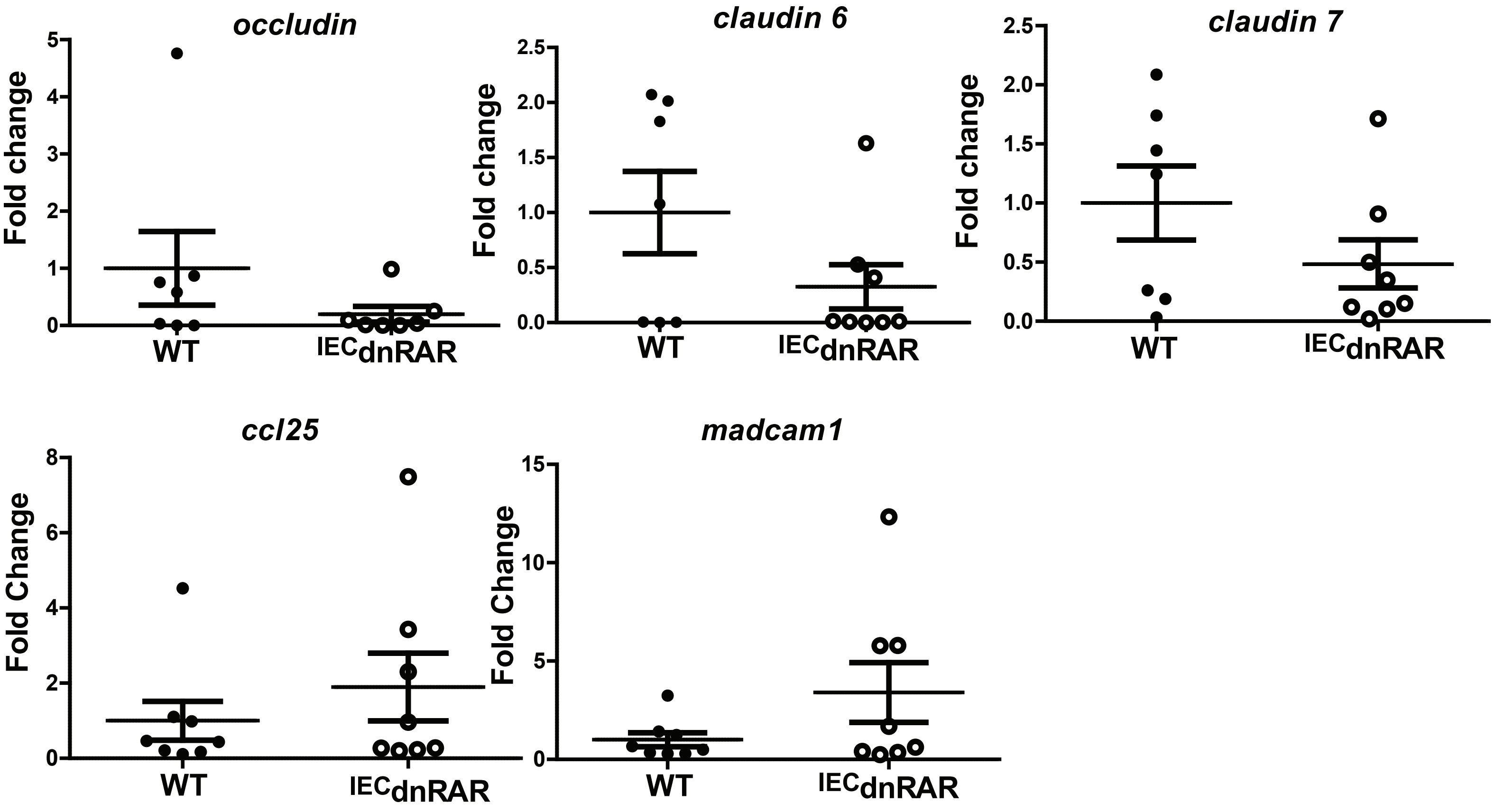

Supplementary figure 3. mRNA expression for *occludin, claudin 6, claudin 7, ccl25 and madcam 1* in the duodenum of the SI in WT and ^{IEC}dnRAR mice . Fold change expression relative to hprt. The mean of the WT was set at 1. Values are the mean ± SEM of two combined experiments and with n=7-8 mice.

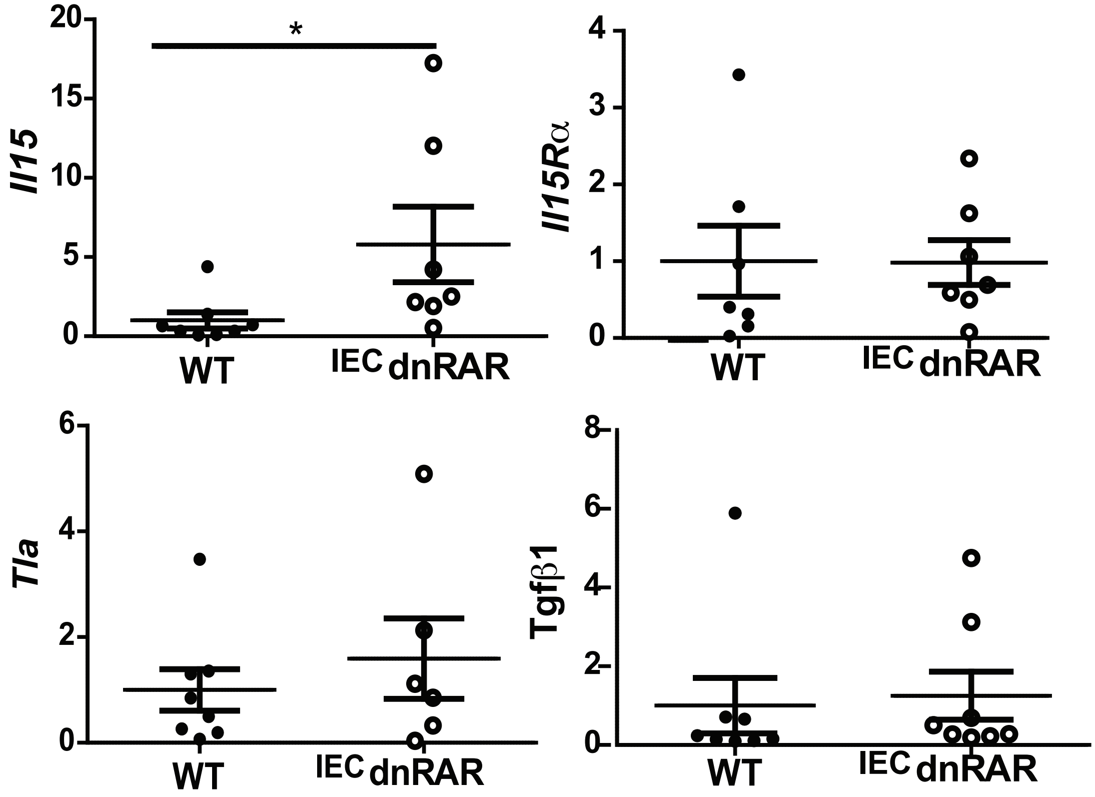

Supplementary figure 4: SI expression of mRNA for IL-15, IL15Rα, TLA and TGFβ1. The doudonem was collected from WT and IEC dnRAR mice. Fold change of *Il15*, *Il15r*α, *Tla* and *Tg*β*1* expression relative to hprt. The mean of the WT was set at 1. Values are the mean ± SEM of two combined experiments and with n=7-8 mice. Significance was determined using the MannWhitney test (A, B, D), or unpaired student's t test (C), *P<0.05.

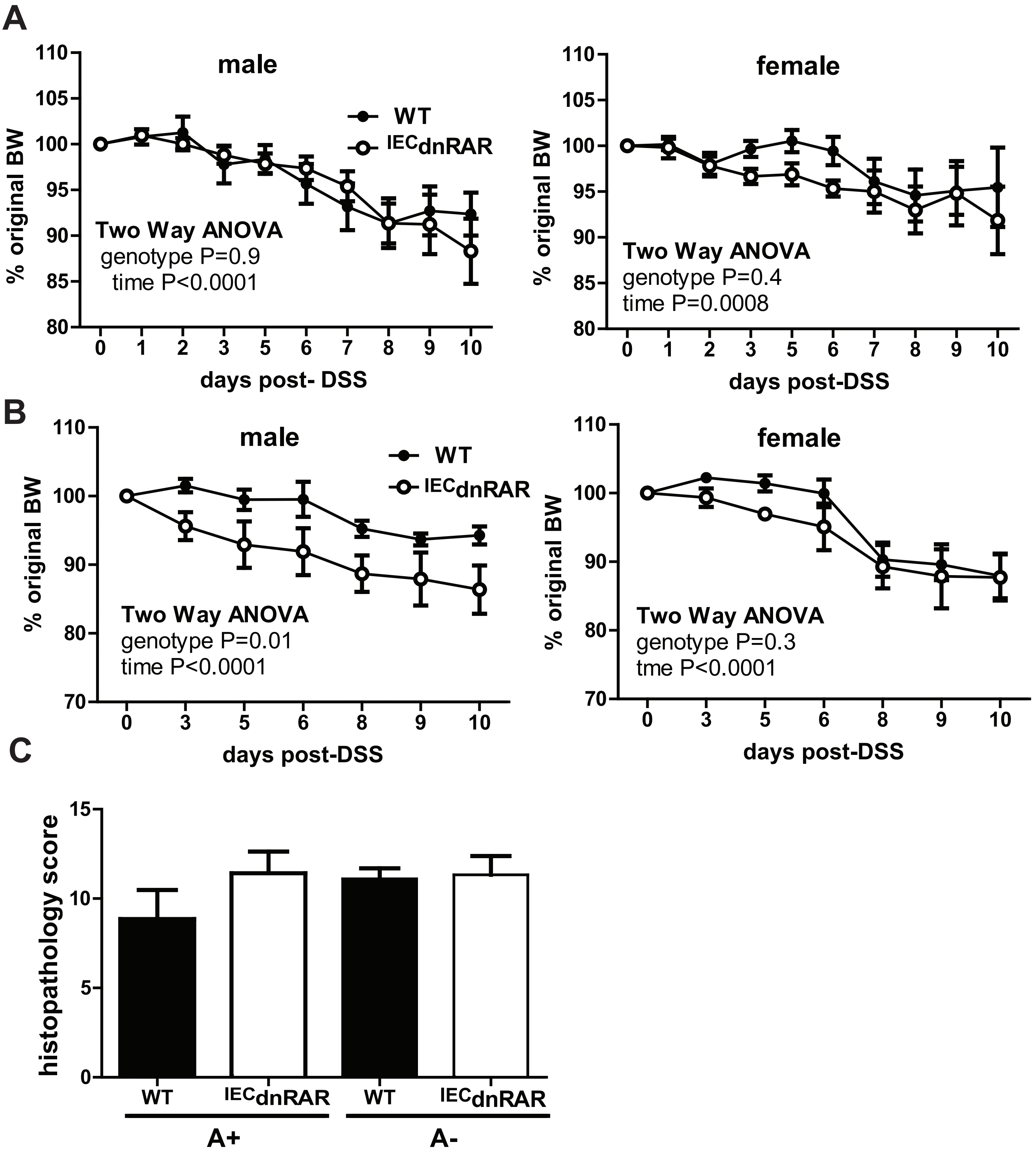

Supplementary figure 5: Susceptibility of A+ and A- WT and IECdnRAR mice to DSS. Percent BW change in **A)** A+ and **B)** A- WT and IECdnRAR male and female mice. **C)** Histopathology scores of A+ and A- mice at d10 post-DSS. Values are the mean + of n= 6-10 mice per group.

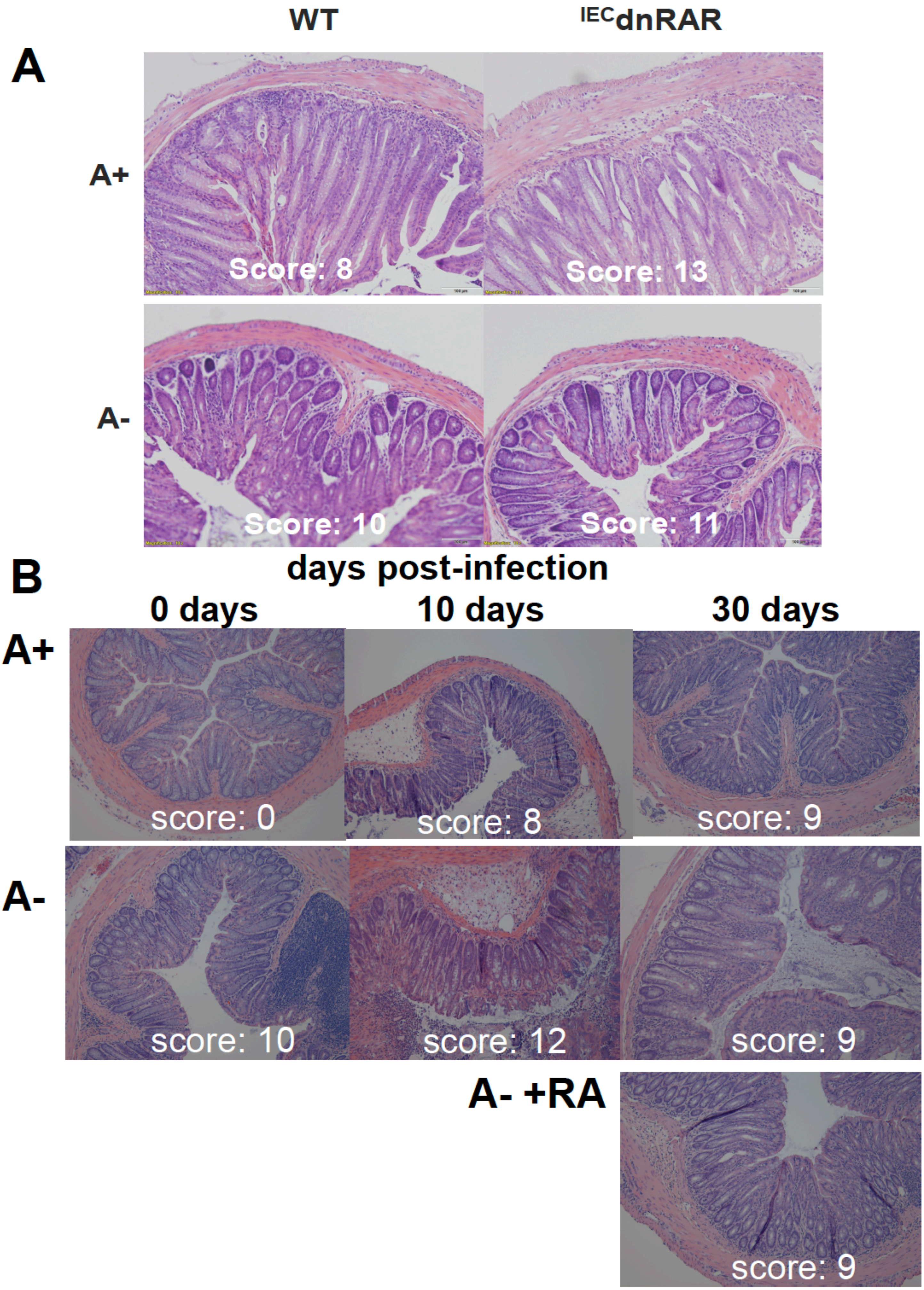

Supplementary figure 6: A) Representative histology images from WT and ^{IEC}dnRAR mice at 10 days post-DSS. The mean histopathology scores are reported in SFig. 5C. **B)** Representative histology images from ^{IEC}dnRAR mice following *C. rodentium* infection. The mean histopathology scores are reported in Fig. 6. Histopathology scores are reported on each section.

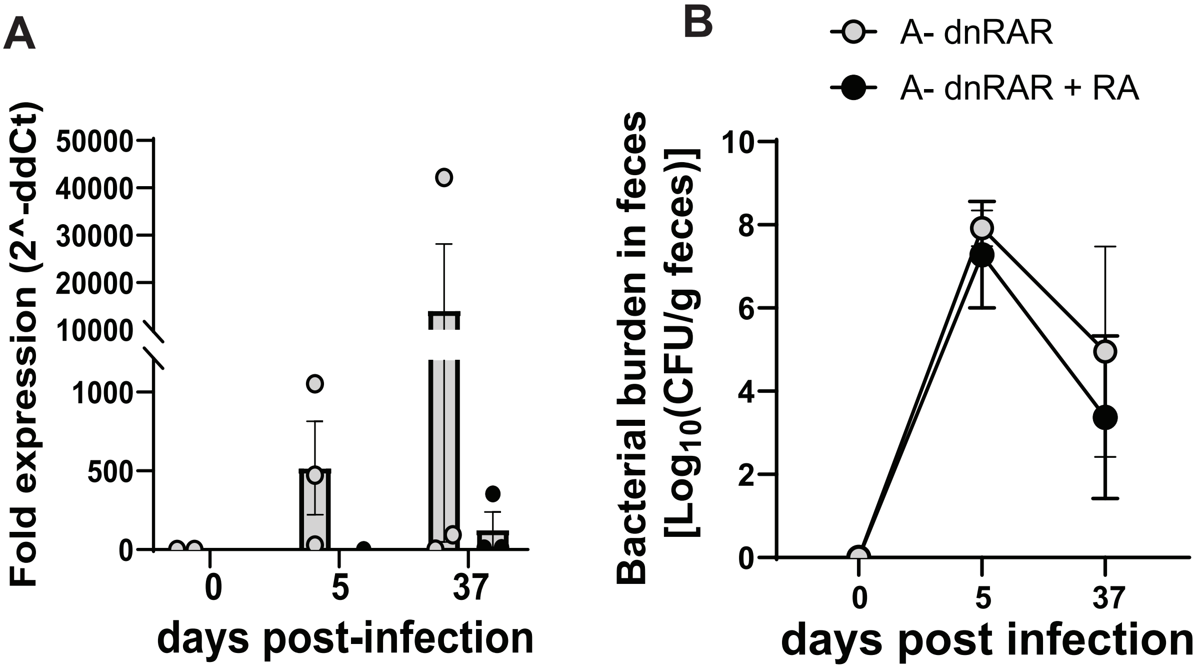

Supplementary figure 7. RA treatment reduces expression of Ler in A- LysMdnRAR mice. A- LysMdnRAR mice were infected with *C. rodentium* with and without RA treatmentt (A- dnRAR and A- dnRAR +RA). **A)** *C. rodentium* Ler fold gene expression was normalized to day 0 values. and **B)** Bacterial burden at days 0, 5 and 37 post *C. rodentium* infection A- vs. RA treated mice. Values are mean ± SEM. n=3-4/group. Multiple t-tests (**A**) and 2-way ANOVA (**B**) were used to test statistical significance. No significant differences were found.
